# Dataset for evaluating the accessibility of the websites of selected Latin American universities

**DOI:** 10.1016/j.dib.2019.105013

**Published:** 2019-12-19

**Authors:** Patricia Acosta-Vargas, Mario González, Sergio Luján-Mora

**Affiliations:** a SI2 Lab, Universidad de las Américas, 170125, Quito, Ecuador; b Department of Software and Computing Systems, University of Alicante, 03690, Alicante, Spain

**Keywords:** Accessibility, Assess, Evaluation, Dataset, Higher education, Website, Web content accessibility guidelines (WCAG) 2.1

## Abstract

This article presents the process of building a dataset for evaluation of the accessibility of 368 web pages, beginning with Webometrics rankings, the WAVE tool was used in the evaluation of the web pages. The dataset documents data on repeated errors with higher frequency, in such a way that they alert the web developers, supporting them in creating more inclusive and accessible websites for all types of people, including users with disabilities. The data show that university websites have frequent problems related to the lack of alternative text linked to images. Some of the university websites included in this dataset were found to violate web accessibility requirements based on the Web Content Accessibility Guidelines 2.0 and 2.1. Therefore, this data has been shared to allow replication of the experiment, and serve as an input to future studies related to web accessibility. The dataset is hosted, with public access, in the Mendeley Dataset Repository.

**Table:** 

**Value of the Data** • The dataset information can help the research community for various applications, such as to predict whether websites are accessible or to determine possible failures in building inclusive website prototypes. It can also be used for clustering analysis or multivariate queries, testing, comparison with similar datasets, and categorization of accessible websites. • These data are useful for knowing the accessibility status of educational websites in Latin America. Some, despite a high ranking, according to Webometrics [1], do not necessarily meet the web content accessibility guidelines defined in the WCAG 2.0 and WCAG 2.1 standards [3]. • On the other hand, these data allow identification of errors repeated with high frequency in the main pages of the 368 websites [4], which can be useful as a reference in the design of more accessible and inclusive websites. • This type of reference data can directly benefit website developers, during design with agile and adaptive methodologies, such that all users, including people with disabilities, can navigate and interact easily on the web. • These data can be compared with outcomes of future evaluations in order to know whether educational institutions have improved their web accessibility, advanced universal access, and raised their visibility in search engines.

## 1 Data

This dataset consists of the data from an evaluation of web accessibility applied to the main pages of Webometrics [1] section Latin American. The dataset is in.xlsx format where each row represents an instance, and each column represents an attribute of the university websites. The multivariate dataset contains 368 instances and 17 attributes. The size of the whole dataset is of 205 Kb. This dataset contains the metadata and supported the analysis for the article published at DOI: 10.1109/ACCESS.2018.2848978.

**Table:** Specifications Table

Subject	Computer Science and Education
Specific subject area	Analysis, Classification Analysis, Web Accessibility
Type of data	Table in.xlsx format Graph
How data were acquired	Web scrapping from Webometrics, automatic evaluation with WAVE (software https://wave.webaim.org/) and manual review by experts.
Data format	Raw, analyzed. The dataset is public and is available in the Mendeley Dataset Repository [2].
Parameters for data collection	The authors performed a web scraping from the Webometrics site. Using an Excel macro, we obtained the URL of each site to evaluate. The URL of each home page was loaded into the Google Chrome browser, and the WAVE plug-in was executed. The resulting data was manually recorded in a spreadsheet that is now stored in the Mendeley Dataset Repository.
Description of data collection	For the evaluation of the main pages of each website, the data was collected as follows. The first phase involved a web scraping of the Webometrics site, in the section of Latin American universities. In the second phase, 368 web pages were randomly selected for evaluation. In phase three, an Excel macro was used to extract each URL and place it in the Google Chrome browser. The WAVE plug-in, version 1.0.9, updated November 17, 2017. WAVE produces a report containing the data and variables involved. Finally, the report data from each web page was manually copied and organized in the spreadsheet.
Data source location	Higher Education Institutions in 26 countries: Antigua Barbuda, Argentina, Aruba, Bolivia, Brazil, Chile, Colombia, Costa Rica, Cuba, Dominica, Ecuador, El Salvador, Guatemala, Haiti, Honduras, Jamaica, Mexico, Nicaragua, Panama, Paraguay, Peru, Puerto Rico, Dominican Republic, Trinidad and Tobago, Uruguay, and Venezuela.
Data accessibility	Mendeley Dataset Repository on https://data.mendeley.com/datasets/526kfj5dpj/1
Related research article	Acosta-Vargas, P., Acosta, T., & Luján-Mora, S. “Challenges to Assess Accessibility in Higher Education Websites: A Comparative Study of Latin America Universities.” *IEEE Access, vol. 6, pp. 36500–36508,* 2018*.* DOI 10.1109/ACCESS.2018.2848978

## 2 Experimental design, materials, and methods

The dataset was compiled by evaluating the accessibility of the randomly selected websites of Latin American universities. Each record contains data, from the website of one institution, based on an automatic quantitative evaluation using WAVE [5]. Using a formula for calculating the sample size, 368 cases were evaluated. The dataset attributes are the characteristics, or variables, determined for each case. The method had four phases.

### 2.1 Phase 1: problem

The work arose from a real need to know if the websites of Latin American universities, which are in the first ranking, according to Webometrics, are accessible. Detailed information on the variables are in Table 1.

**Table 1:** Description of dataset variables.

Name	Description	Type
University	It is the name of the University taken in the case study.	Text
URL	It is the website address of the university.	Text
Acronym	It is the short name defined for the university.	Text
Country	The variable indicates the country name of the educational institution.	Text
Latin America Ranking	It is the numeric value assigned by the webometrics institution according to the location in the ranking of higher education institutions for Latin America.	Numeric
World Ranking	It is the numerical value assigned by the webometrics institution according to the location in the ranking of higher education institutions for the whole world.	Numeric
Presence	This variable is the number of web pages of the main web domain of the institution. It includes all subdomains and all file types, including pdf documents.	Numeric
Impact	This value represents the external networks (subnets) that create backlinks to the institution's web pages. After normalization, the average value between the two sources is selected. This variable is related to the visibility of the website.	Numeric
Opening	This variable is related to the number of citations of the principal authors, according to the Google Scholar citations source.	Numeric
Excellence	This variable relates to the number of academic articles published in high-impact international journals in the top 10% of their respective scientific disciplines. The data provider is the SCimago Group.	Numeric
Errors	A variable defined by WAVE indicates that it detected an error. The absence of errors does not mean that a page is accessible. Red icons indicate accessibility errors that need to be corrected.	Numeric
Alerts	Indicates the elements that evaluators observe that represent a problem for the end-user.	Numeric
Features	Indicate accessibility features, things that are likely to improve accessibility, but that need to be verified.	Numeric
Structural Elements	They represent the alerts that the evaluators must review in the structure of the web page.	Numeric
HTML5 and ARIA	This variable is defined by WAVE and represents the web accessibility errors that the evaluator must correct on how to add accessibility information to HTML elements using the Accessible Rich Internet Applications specification.	Numeric
Contrast Errors	Represents the alerts that evaluators should review in the Errors of Contrast section.	Numeric

### 2.2 Phase 2: data compilation

The experimental process began by navigating to the main page of each website and evaluating with WAVE [6] using the following process (1) install the WAVE plug-in for Google Chrome, (2) enter the Google Chrome browser, (3) type the URL of the website to be evaluated, (4) load the page, (5) click on the installed plug-in, (6) obtain the data, and (7) record the data obtained in a spreadsheet. The WAVE web accessibility assessment tool had been used in previous studies by the authors [4,6,7]. The tools are not a panacea for accessibility issues and always require interpretation by an expert in web accessibility.

### 2.3 Phase 3: cleaning and homogenizing the data

In this phase, it was essential to apply an appropriate format to each variable. In this case, quantitative variables we used. (1) Data analysis: web scrapping was initially applied to extract the Webometrics web to Excel. After extracting the data, the experts carried out a manual inspection of the data sample to detect data quality problems that might affect its properties. (2) Definition of the transformation flow: Using macros the URL of each website was extracted; several Excel functions were used to corrected errors of accents and spaces. (3)Verification: we applied, through multiple iterations, the steps of analysis, design, and verification. Some errors only became evident after applying a certain number of transformations to the data. (4) Clean data flow: once the quality errors have been eliminated, the clean data were used to perform the analysis.

### 2.4 Phase 4: graphics, data analysis, and discussion

In this phase, graphs were made to identify the relationships that exist between the variables, in a way that we could predict the behavior of the websites of Latin American universities. This dataset formed part of the data analyzed in an article related to the challenges of web accessibility for Latin American universities [4].

**Fig. 1:**
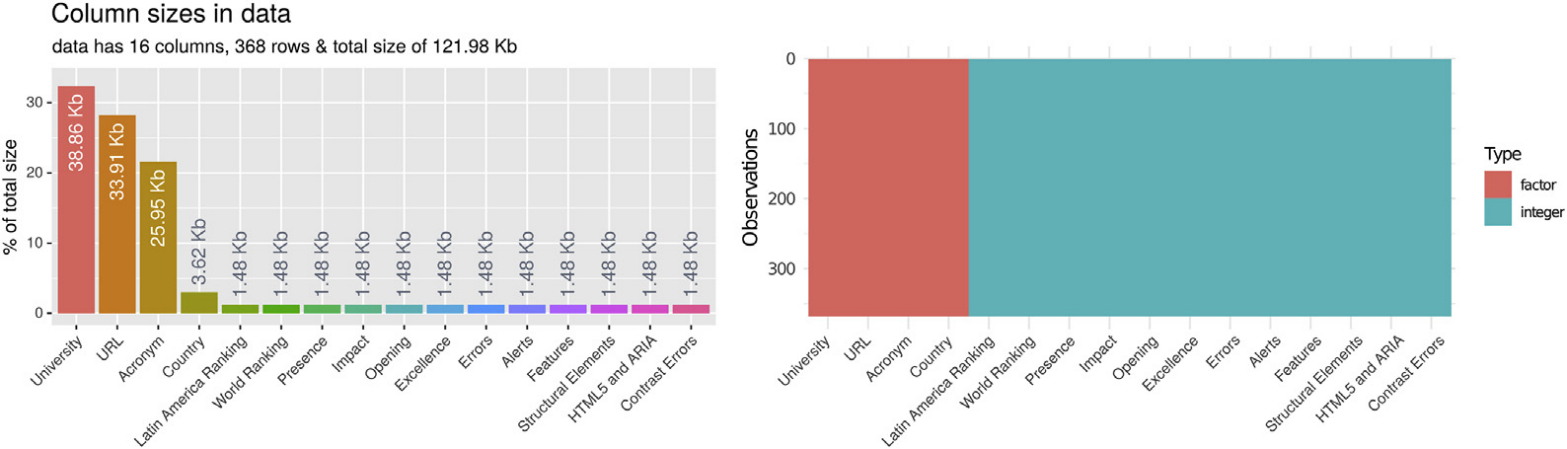
Data columns sizes and types.

Fig. 1-left depicts the size in Kb of the different columns in the dataset. As expected, the factor variables (strings) take up a larger size in memory than the numerical variables. Fig. 1-right depicts the variable types. University, URL, Acronym, and Country are factor variables; and Latin America Ranking; World Ranking; Presence, Impact, Opening, Excellence, Errors, Alerts, Features, Structural Elements, HTML 5 and Aria, and Contrast Errors are numerical (integer) discrete variables.

**Fig. 2:**
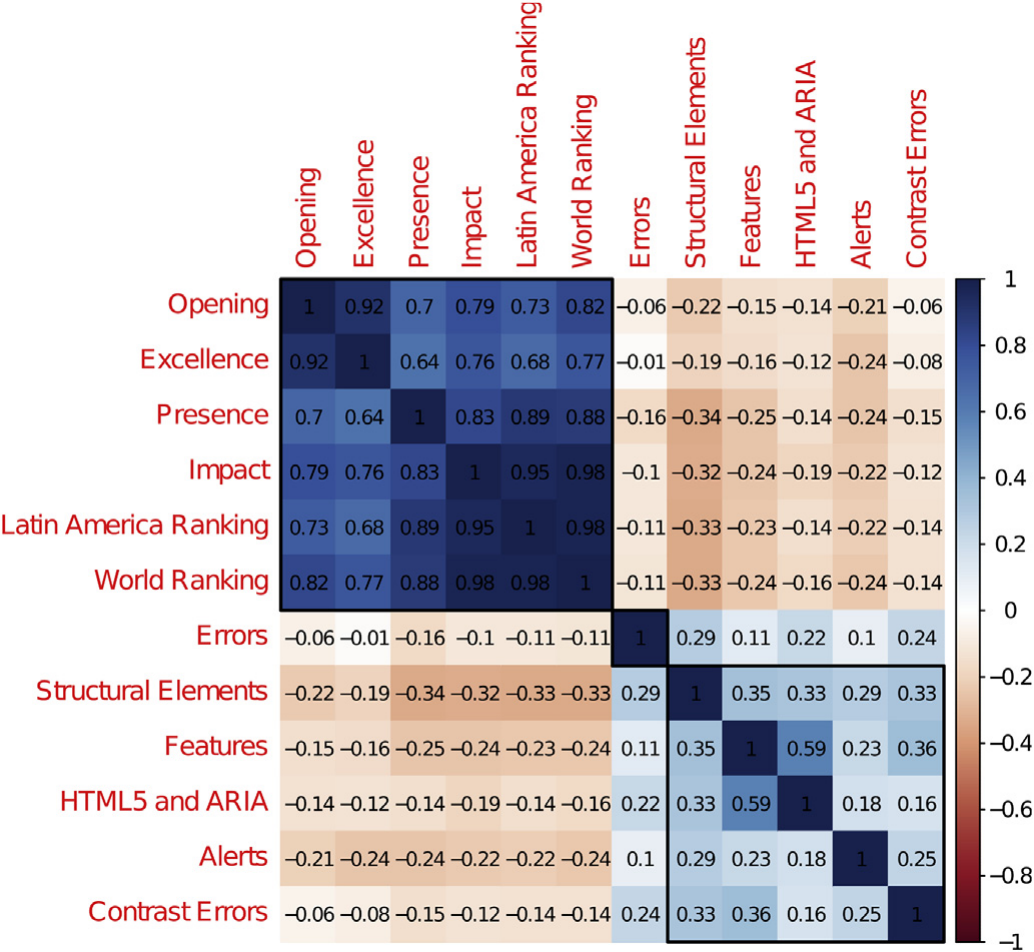
Correlation for numeric variables.

Fig. 2 shows the correlation among the numerical variables. Three groups were defined according to the correlations between the variables. All variables related to the Webometrics [1] rankings belong to the same group. The variables corresponding to the output of the WAVE accessibility evaluation (except Errors) form the second category: Structural Elements, Features, HTML5 and ARIA, Alerts, and Contrast. The variable Errors remains alone; Errors is a critical variable among the accessibility data. From Fig. 2, it is evident that its relationship with other WAVE evaluation variables is not trivial.

**Fig. 3:**
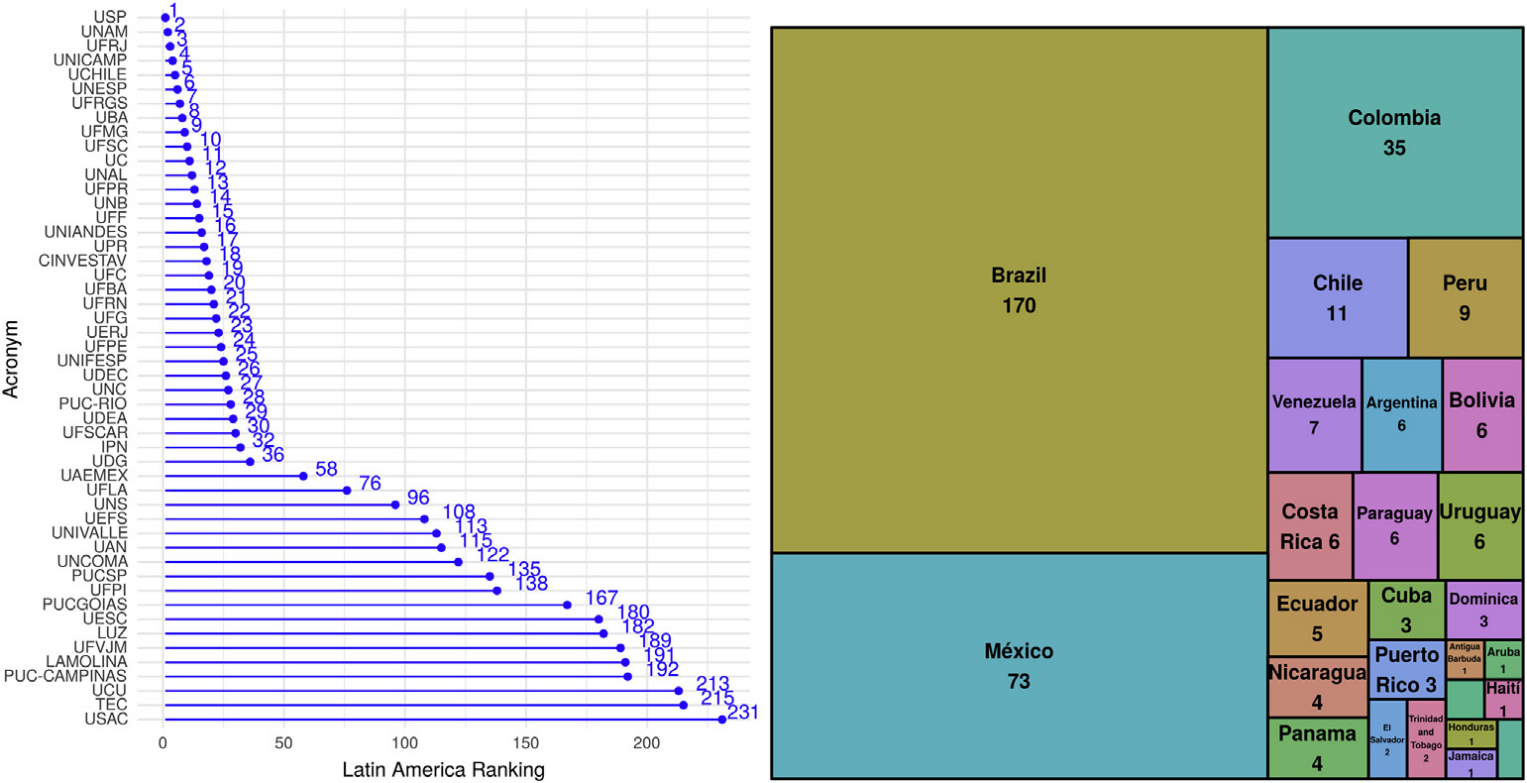
Right: The top 50 universities in the dataset ranked. Left: Number of universities in the dataset by country.

The dataset contains information on 368 websites from Webometrics. The top 50 universities are represented in Fig. 3- left. The countries of origin present in the dataset and their importance in terms of appearance are shown in Fig. 3- right, with Brazil, Mexico, Colombia, Chile, and Peru the countries with the most institutions in the dataset.

## Transparency document

A transparency document associated with this article can be found in the online version at https://doi.org/10.1109/ACCESS.2018.2848978.
